# Acute molecular responses to concurrent resistance and high-intensity interval exercise in untrained skeletal muscle

**DOI:** 10.14814/phy2.12364

**Published:** 2015-04-22

**Authors:** Jamie K Pugh, Steve H Faulkner, Andrew P Jackson, James A King, Myra A Nimmo

**Affiliations:** 1 School of Sport, Exercise and Health Sciences, Loughborough UniversityLoughborough, UK; 2 College of Life and Environmental Sciences, University of BirminghamBirmingham, UK

**Keywords:** Acute responses, cellular signaling interference, concurrent exercise, high-intensity interval training, human skeletal muscle

## Abstract

Concurrent training involving resistance and endurance exercise may augment the benefits of single-mode training for the purpose of improving health. However, muscle adaptations, associated with resistance exercise, may be blunted by a subsequent bout of endurance exercise, via molecular interference. High-intensity interval training (HIIT), generating similar adaptations to endurance exercise, may offer an alternative exercise mode to traditional endurance exercise. This study examined the influence of an acute HIIT session on the molecular responses following resistance exercise in untrained skeletal muscle. Ten male participants performed resistance exercise (4 × 8 leg extensions, 70% 1RM, (RE)) or RE followed by HIIT (10 × 1 min at 90% HR_max_, (RE+HIIT)). Muscle biopsies were collected from the *vastus lateralis* before, 2 and 6 h post-RE to determine intramuscular protein phosphorylation and mRNA responses. Phosphorylation of Akt (Ser^473^) decreased at 6 h in both trials (*P *<* *0.05). Phosphorylation of mTOR (Ser^2448^) was higher in RE+HIIT (*P *<* *0.05). All PGC-1*α* mRNA variants increased at 2 h in RE+HIIT with PGC-1*α* and PGC-1*α*-ex1b remaining elevated at 6 h, whereas RE-induced increases at 2 and 6 h for PGC-1*α*-ex1b only (*P *<* *0.05). Myostatin expression decreased at 2 and 6 h in both trials (*P *<* *0.05). MuRF-1 was elevated in RE+HIIT versus RE at 2 and 6 h (*P *<* *0.05). Atrogin-1 was lower at 2 h, with FOXO3A downregulated at 6 h (*P *<* *0.05). These data do not support the existence of an acute interference effect on protein signaling and mRNA expression, and suggest that HIIT may be an alternative to endurance exercise when performed after resistance exercise in the same training session to optimize adaptations.

## Introduction

It is well established that different types of training programs result in distinct physiological adaptations in response to different stimuli and environmental changes (Haddad and Adams 2002). Divergent exercise modes develop different phenotypes, and when undertaken concurrently, endurance exercise may interfere with the adaptation of a single-mode resistance exercise delivery. Work exists to both support (Hickson 1980; Coffey et al. 2009a,b) and refute (Donges et al. 2012; Lundberg et al. 2012; Apró et al. 2013) the suggestion that concurrent training results in molecular interference and attenuated strength development. Despite this, exercise guidelines recommend individuals perform a combination of resistance and endurance exercise to improve cardio-metabolic health and quality of life (Chief Medical Office 2011; Garber et al. 2011). As lack of time is often cited as a reason for individuals not meeting current physical activity guidelines (Stutts 2002; Trost et al. 2002), an exercise program that combines both exercise modes within a single exposure, which does not interfere with the effects of the prior resistance work may benefit exercise adherence. In order to avoid potential interference of the low intensity, long duration endurance exercise, and alternative protocol, high-intensity interval training (HIIT), may be a beneficial substitute. HIIT has recently been suggested as a time-efficient alternative (<25 min) to traditional endurance exercise with profound increases in V̇O_2 max_ and glucose tolerance (Gibala et al. 2006; Burgomaster et al. 2008). Therefore, an alternative combination of exercise, that is, resistance exercise plus HIIT, could gain the health and quality of life outcomes required, but only involve limited training time.

In recent years, information detailing the molecular signaling pathways that mediate these divergent training adaptations have demonstrated an association between acute bouts of resistance exercise and the activation of the mechanistic target of rapamycin complex 1 (mTORC1) pathway, which modulates protein synthesis and over time can result in hypertrophy (Egan and Zierath 2013) through improvement in translation efficiency (Dreyer et al. 2010). Downstream phosphorylation of p70S6K-rpS6 and 4E-BP1 pathways promote protein synthesis through translation initiation (Bodine et al. 2001; Léger et al. 2006). Protein synthesis is also regulated by the dephosphorylation of eEF2, thereby enhancing translation elongation (Browne and Proud 2002; Kapp and Lorsch 2004). Increased activity in these proteins could indirectly demonstrate enhanced protein synthesis. Conversely, activation of AMPK and p38 mitogen-activated protein kinase during endurance exercise has been shown to regulate PGC-1*α* and result in endurance-type adaptation (Arany 2008). PGC-1*α* may stimulate mitochondrial biogenesis, and has been linked to endurance adaptation (Olesen et al. 2010). However, Ruas et al. (2012) suggested that preferential expression of PGC-1*α* splice variants may occur with endurance and resistance exercise. A truncated PGC-1*α* splice variant (termed PGC-1*α*4 by Ruas et al.), originating from the alterative promoter (exon 1b derived), was linked with muscle hypertrophy through increases in insulin-like growth factor (IGF)-1 and the suppression of myostatin. Conversely, full-length PGC-1*α* (termed PGC-1*α*1 by Ruas et al. 2012) originating from the proximal promoter (exon 1a derived), was suggested to be responsible for more aerobic adaptation. This gives rise to the possibility of an increase in total PGC-1*α* expression following both modes of exercise via different signaling pathways.

The interference noted with concurrent training may involve the activation of AMPK after endurance exercise (McGee and Hargreaves 2010) which can inhibit the Akt-mTOR cascades activated by resistance exercise, and consequently suppress muscle growth (Hickson 1980; Coffey et al. 2009a,b). Studies have shown no interference effect with concurrent exercise where the endurance and resistance exercise components have been either separated by a matter of hours (Lundberg et al. 2012), or exercise modes were not evenly matched (Apró et al. 2013). However, when concurrent exercise is completed within a single session Coffey et al. (2009a,b) found diminished anabolic responses to resistance exercise when it was preceded by endurance exercise, whereas increased muscle protein degradation and inflammation were observed when resistance exercise was completed prior to endurance exercise (Coffey et al. 2009b) but there was no detrimental effect on the anabolic response. In a later study (Coffey et al. 2009a), these responses were exaggerated when the endurance exercise was replaced by supra-maximal repeated sprints consisting of 10 × 6 sec ‘all out’ sprints with 54 sec recovery. This suggests that an exercise protocol where endurance exercise comes after resistance is the preferred option to optimize the anabolic responses but could lead to greater local muscular damage, evidenced by enhanced inflammation and proteolytic activity (Coffey et al. 2009a,b). Also the exaggeration response with high-intensity exercise suggests that an intensity below maximal might be advantageous. In addition, the previous repeated, ‘all out’, supra-maximal sprints are extremely demanding, requiring high levels of motivation as well as causing feeling of severe fatigue, which may not be well suited or practical for all populations (Coyle 2005). The use of more tolerable HIIT models, such as the 10 × 1 min at 90% of maximal heart rate (HR_max_) used by Little et al. (2010), may be better suited to untrained, less motivated individuals.

Often in the literature the acute molecular responses to concurrent exercise are examined in a fasted state, yet a large percentage of individuals spend the majority of the day in a postprandial state (Lopez-Miranda et al. 2007). Furthermore, the influence of feeding on the activation of anabolic signaling (Cuthbertson et al. 2005; Fujita et al. 2007) has been shown to be independent of the resistance exercise (Dreyer et al. 2006; Eliasson et al. 2006) which together further enhance mTOR signaling (Koopman et al. 2007; Dreyer et al. 2008) and muscle protein synthesis (Tipton et al. 1999; Miller et al. 2003). Therefore, in the present study, the resistance exercise session has been designed in accordance with the ACSM guidelines for untrained individuals (Kraemer et al. 2009), along with a practical, time-efficient HIIT protocol combined in a single session, performed after a nutritionally balanced breakfast to establish further understanding of whether an interference effect exists in a fed “real-life” training state.

The aim of this investigation was to examine the effect of a single session of high-intensity interval cycling immediately following lower-body resistance exercise on the acute molecular responses compared to a single bout of resistance exercise on the exercise-specific muscle adaptations. It was hypothesized that the translational machinery (mTORC1 pathway) and mRNA expression related to skeletal muscle growth would not be interfered with when performing concurrent resistance exercise followed by HIIT, in an untrained population, compared to an isolated resistance exercise session.

## Methods

### Participants

Ten healthy male participants were recruited to participate in this study (age; 21.3 ± 1.0 years, height; 1.80 ± 0.02 m, mass; 76.2 ± 3.6 kg, waist circumference; 79.3 ± 2.1 cm) without any structured exercise training for the last 12 months. Maximal strength of the quadriceps was 59.1 ± 3.4 kg (range: 47.5–76.3 kg). 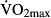 was 44.9 ± 1.8 mL kg min^−1^ (range: 37.5–58.0 mL·kg·min^−1^). All participants were nonsmokers, free from injury and not taking any medication or nutritional supplements. All participants provided full written informed consent. The Human Research Ethics Committee of Loughborough University approved all study procedures.

### Study design

**Figure 1:**
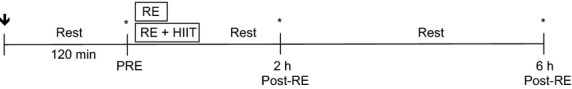
Schematic diagram of the experimental trials. This study adopted a counterbalanced crossover design where participants completed both exercise trials on separate occasions. RE, resistance exercise trial; RE + HIIT, resistance exercise and high-intensity interval training trial. Arrow indicates time of breakfast. * indicate sampling time points for muscle biopsies.

This study adopted a counterbalanced crossover design (Fig.1). In one trial participants completed an acute resistance exercise session only (RE) and in the other trial participants performed RE followed by a HIIT session (RE + HIIT), each trial was separated by a minimum of 7 days (range: 7–25 days), during which time the participants were instructed to maintain their habitual lifestyle.

### Preliminary testing

#### Maximal strength

Participants were asked to arrive fasted (at least 4 h) and having avoided any strenuous physical activity 48 h before the preliminary tests. Each participant performed a unilateral one-repetition maximum (1RM) on each leg using a leg extension machine (Technogym, Cesena, Italy). Participants were familiarized with the movement and warmed up prior to testing by performing six repetitions (at ∼40% of estimated 1RM) and four repetitions (at ∼60% of estimated 1RM) through a full range of motion with 1 min rest. After each successful lift the weight was increased until a failed attempt occurred with 3 min recovery between each attempt. The 1RM was attained within five attempts.

#### 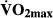


Following a 30 min rest, a continuously ramped 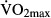 test was performed on an electrically braked cycle ergometer (Lode Excalibur, Groningen, The Netherlands). Following a 5 min warm up at 50 W, workload progressively increased at 20.5 W·min^−1^ until the participant reached volitional exhaustion. Oxygen consumption (V̇O_2_) was obtained through breath-by-breath sampling (Cortex Metayzer 3B, Leipzig, Germany) that was calibrated prior to each test using gases of known concentrations (17.10% O_2_ and 5% CO_2_) and a 3 L Hans Rudolph syringe. 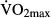 was determined as the highest value achieved over an 11 breath average. Heart rate was continuously recorded during the exercise (RS300, Polar, Finland) and participants were asked to maintain a cadence between 80–100 r·min^−1^. Participants performed a familiarization session to the RE and HIIT one week before the first experimental trial.

### Diet and physical activity control

Participants were instructed to avoid alcohol, caffeine and physical activity during the 48 h period prior to the two main experimental trials. Diet and physical activity levels were recorded for 24 h before the first experimental trials and participants were asked to replicate dietary intake and physical activity prior to the second experimental trial. A standardized breakfast was provided for the participants on the morning of each trial, 2 h before the baseline skeletal muscle biopsy. The breakfast (1803 ± 46 kJ) was nutritionally balanced (55% carbohydrate; 29% fat; 16% protein) and provided 15% of the participant's estimated recommended daily energy intake based on their calculated BMR (Mifflin et al. 1990) and a physical activity level of 1.6 (lightly active to sedentary lifestyle).

### Experimental trials

Participants arrived at the laboratory at ∼0730 following an overnight fast (∼10 h) and consumed the standardized breakfast. After finishing the meal, participants remained at rest for 2 h before a resting skeletal muscle biopsy was taken using an automatic biopsy needle (11G ACECUT, TSK Laboratory, Europe B.V.). Local anesthesia (2 mL, 1% lidocaine) was administrated into the subcutaneous tissue of the *vastus lateralis*. Two muscle samples of ∼30 mg were obtained, with visible fat and excess blood removed. Samples were then immediately frozen in liquid nitrogen and stored at −80°C until subsequent analysis. Participants then performed either RE or RE + HIIT. During both exercise sessions, participants received continuous verbal encouragement. For all trials, ratings of perceived exertion (RPE; Borg CR10 scale) (Borg 1998) were recorded after each set of individual leg extensions and each 1 min repetition of high-intensity cycling. Subsequent muscle biopsies were taken 2 and 6 h post-RE. Participants remained in the laboratory, fasted and were allowed to consume water ad libitum throughout.

### Resistance exercise (RE)

Participants completed a standardized warm up consisting of two sets of eight repetitions of unilateral leg extensions at 30% 1RM, immediately followed by the contralateral leg. This was followed by four sets of eight repetitions at 70% 1 RM on each leg. All repetitions were matched for velocity and range between trials, and each set was separated by a 2 min passive recovery period.

### High-intensity interval training (HIIT)

Immediately after the 2 min recovery following the final RE set, participants completed a 3 min standardized warm up at 50 W on the cycle ergometer. This was followed by the completion of 10 repetitions of 1 min cycling at an intensity designed to elicit 90% of HR_max_, with each repetition separated by 1 min of cycling at 50 W. Participants were instructed to maintain a cadence between 80–100 r·min^−1^.

### Western blot analysis

Skeletal muscles samples were homogenized at 20 Hz for 2 × 3 min using a TissueLyser II (Qiagen, Hannover, Germany) in 300 *μ*L of ice-cooled buffer (1 × PBS containing 1% Triton X-100, 1% protease and phosphatase inhibitor cocktail [Thermo Scientific, Rockford, IL; cat. 1861281], and 1% 0.5 M EDTA [Thermo Scientific; cat. 1861274]). Supernatants were aliquoted following centrifugation of homogenates at 17,000 × *g* for 10 min at 4°C. Protein concentrations were determined using a Pierce 660 protein assay kit (Pierce Biotechnology, Rockford, IL). After protein determination, NuPAGE LDS sample buffer (Invitrogen, Carlsbad, CA), *β*-mercaptoethanol and distilled water were added to the protein samples at a final protein concentration of 0.75 *μ*g·*μ*L^−1^, vortexed and boiled at 95°C for 5 min to denature proteins. Samples were stored at −20°C for no longer than 14 days before subsequent analysis.

Prepared protein samples were separated by SDS-PAGE on NuPAGE 10% Bis-Tris gels (Invitrogen). Electrophoresis was performed in ice-cooled NuPAGE MOP SDS running buffer (Invitrogen) at 125 volts for 2 h. The transfer of proteins to polyvinylidine fluoride membrane was then performed at 30 volts for 1 h in ice-cooled NuPAGE transfer buffer (Invitrogen) with 10% methanol. Membranes were blocked with gentle agitation for 1 h at room temperature in Tris-buffered saline (TBS; 50 mmolL^−1^ Tris base, 150 mmolL^−1^ NaCl, pH 7.6) containing 0.05% Tween-20 (TBST), with either 5% BSA or 5% nonfat dry milk depending on the primary antibody of interest (see below). After blocking, membranes were incubated with gentle agitation overnight at 4°C in primary antibodies and the appropriate TBST blocking buffer.

All antibodies were purchased from New England BioLabs (Hitchin, Hertfordshire, UK). Anti-rabbit primary antibodies were used to detect changes in phosphorylation of Akt on Ser^473^ (monoclonal; no. 4060), mechanistic target of rapamycin (mTOR) at Ser^2448^ (monoclonal, no. 5536), p70 S6 kinase (p70S6K) at Thr^389^ (monoclonal, no. 9234), 4E-binding protein-1 (4E-BP1) at Thr^37/46^ (monoclonal, no. 2855), eukaryotic elongation faction 2 (eEF2) at Thr^56^ (polyclonal, no. 2331) and ribosomal protein S6 (rpS6) at Ser^235/236^ (monoclonal, no. 4858). Anti-rabbit primary antibodies for *α*-tubulin (monoclonal, no. 2125) were used to correct for any differences in protein volume in each sample. The primary antibodies for p70 S6K, 4E-BP1, rpS6, *α*-tubulin were diluted 1:1000 in TBST composing of 5% nonfat dry milk. The primary antibodies for Akt, mTOR, and eEF2 were diluted 1:2000 in TBST composing of 5% BSA.

The membrane was washed four times (3 × 5 min, 1 × 15 min) in TBST before incubation with the secondary antibody for 90 min at room temperature. Secondary anti-rabbit IgG, HRP-linked antibody (polyclonal, no. 7074) was diluted 1:2000 in TBST composing of the same blocking solution as the primary antibody of interest. Membranes were then washed in TBST (3 × 5 min, 1 × 15 min). Finally, detection of proteins were made on a Molecular Imager ChemiDoc XRS+ imaging system (Bio-Rad Laboratories, Richmond, CA) via chemiluminescence using SuperSignal West Dura Chemiluminescent substrate (Thermo Scientific). Quantification of band intensities were analyzed using the band detection tool in Quantity One software (version 4.6.8; Bio-Rad Laboratories). All samples (6 biopsy samples) related to each participant were run on the same gel. All samples are expressed relative to *α*-tubulin expression levels. Preliminary testing showed excellent linearity in the loading control protein (*α*-tubulin) over the range (1–25 *μ*g, *r*^2^ = 0.999). All protein loads were run in triplicates (11.7 ± 1.4% coefficient of variation).

### RNA extraction and real-time quantitative PCR

Skeletal muscles samples were homogenized at 20 Hz for 2 × 3 min using a TissueLyser II (Qiagen) in 1.0 mL of ice-cooled TRIzole Reagent (Invitrogen). Following centrifugation at 13,000 × *g* for 15 min at 4°C the supernatant was incubated for 5 min at room temperature. Next, 200 *μ*L of chloroform was added and vortexed for 20 sec then allowed to stand for a further 10 min at room temperature before centrifugation. The upper, clear, aqueous phase containing total RNA was mixed with 1 volume of isopropanol and incubated for 30 min at room temperature before further centrifugation. The RNA pellet was washed in 1.0 mL of ice-cooled 70% ethanol, again centrifuged and then repeated, before air drying. Precipitated RNA was then resuspended in diethylpyrocarbonate-treated water and 1.0 *μ*L of each RNA sample was analyzed on a NanoDrop 2000 UV-Vis Spectrophotometer (Thermo Scientific) to determination RNA concentration and purity. Mean RNA concentration was 125.7 ± 7.6 ng·*μ*L^−1^, and the A_260_/A_280_ ratio, as a measure of purity was 1.83 ± 0.02. An Agilent 210 Expert Bioanalyzer with RNA 6000 Pico LabChip kits (Agilent Technologies, Palo Alto, CA) was used to analyze the size and distribution of extracted RNA molecules. Subsequently, an RNA integrity number (RIN) was calculated for all samples based on the RIN algorithm of the Agilent 2100 Expect software (version B.02.08). Mean RIN was 6.3 ± 0.1. Reverse transcription of 20 *μ*L of cDNA was performed using 2 *μ*g of RNA with a high-capacity RNA-to-cDNA kit (Invitrogen). The cDNA samples were then stored at −20°C until further analysis.

Quantitative real-time PCR (qPCR) was performed on a ViiA 7 Real-Time PCR system (Applied Biosystems) under the following PCR cycle conditions; 50°C for 2 min + 95°C for 10 min + ((95°C for 15 sec + 60°C for 1 min) × 40 cycles). PCR reactions with 2 × TaqMan Universal Master Mix II with UNG (Invitrogen) and 20 × TaqMan Gene Expression assays (Invitrogen) according to the manufacturer's instructions were used to determine mRNA expression levels for myostatin (Hs00976237_m1), muscle RING-finger protein-1 (MuRF-1, Hs00261590_m1), atrogin-1 (Hs01041408_m1), forkhead box 03A (FOXO3A, Hs00818121_m1), myogenic differentiation 1 (MyoD1, Hs00159528_m1), myogenin (MyoG, Hs00231167_m1), *β*-2-microglobulin (*β*2M, Hs00984230_m1), *β*-actin (Primer Design, Southampton, UK), and DNA topoisomerase 1 (TOP1, Primer Design). In addition, PCR reactions with 2 × SYBR Green JumpStart Taq Ready Mix (Sigma-Aldrich), forward and reverse primers (Sigma-Aldrich) at 500 nmol·L^−1^ were used to determine the mRNA expression levels for PGC-1*α* total and splice variants, IGF-1 and mechano-growth factor (MGF). SYBR Green primer sequences are shown in Table1. A melt curve was run on all SYBR Green PCR reactions to assess the amplification specificity. Primers for identifying total PGC-1*α* were located in exon 2. Forward primers for the PGC-1*α* proximal promoter splice variant (termed PGC-1*α*-ex1a) covered exon 1a and exon 2 boundaries, with the reverse primer located in exon 2. Forward primers for the alternative promoter (termed PGC-1*α*-ex1b) was located in the exon 1b insert, with the reverse primer spanning exon 1b and 2 boundaries. All samples were run in triplicates, and all samples from each participant were run together on the same plate to allow for relative comparison. Data were analyzed by cycle threshold values, calculating relative expression using the ΔC_T_ method. Gene expression was normalized using the geometric mean of three reference genes (*β*2M, *β*-actin, TOP1). geNorm software analysis in qBase ^plus^ (Biogazelle, Belgium) was used to test the stability of the reference genes against the protocol used in the present study to determine the optimum number of reference genes.

**Table 1:** SYBR Green primers set sequences

Target mRNA	Strand	Primer Sequence (5′ → 3′)	Amplicon size (bp)
*PGC-1α total*	Forward	cagcctctttgcccagatctt	99
	Reverse	gtggactcaagtggtgcagt	
*PGC-1α-ex1a*	Forward	atggagtgacatcgagtgtgct	127
	Reverse	acagctttctgggtggactc	
*PGC-1α-ex1b*	Forward	tcacaccaaacccacagaga	61
	Reverse	ctggaagacatgatacac	
*IGF-1*	Forward	ggctgaccaagctgaaactc	176
	Reverse	acctcctgggtttaagcgat	
*MGF*	Forward	ggctgaccaagctgaaactc	176
	Reverse	acctcctgggtttaagcgat	

PGC-1α, peroxisome proliferator-activated receptor gamma coactivator 1-alpha; IGF-1, insulin-like growth factor 1; MGF, mechano-growth factor; bp, base pair.

### Statistical analysis

Data were analyzed using IBM SPSS version 21 statistical software (IBM, Chicago, IL). For missing data points (RE + HIIT trial at 6 h in one participant), mean substitutions were used in the analysis. A within-participant, one-way repeated measure ANOVA was used to analyze RPE responses to the exercise trials. A within-participant, two-way repeated-measures ANOVA (trial × time) was used to identify changes in phosphorylation status of all measured signaling proteins data (mTOR, Akt, eEF2, p70 S6K, rpS6, and 4E-BP1) and all measured gene expression data (PGC-1*α* total, PGC-1*α*-ex1a, PGC-1*α*-ex1b, myostatin, MuRF-1, atrogin-1, FOXO3A, IGF-1, MGF, MyoD1, and MyoG). When a main effect of trial or time or interaction was identified a pairwise multiple comparisons with a Bonferroni correction was used to locate differences. Differences in all data sets were considered statistically significant at *P *<* *0.05. Data are expressed as mean ± standard error (SE).

## Results

### Exercise trial responses

All participants completed the same number of sets and repetitions (4 sets × 8 repetitions at 70% 1RM; 39.8 ± 2.5 kg). Similarly, no differences for overall RPE scores (*P *>* *0.05) were observed between exercises in both trials with RE and RE + HIIT being rated as equally strenuous (RE only, 5.5 ± 0.3; RE + HIIT, 5.5 ± 0.3; [RE component, 5.2 ± 0.3; HIIT component, 5.8 ± 0.4]). Average heart rate during HIIT intervals corresponded to 90 ± 2% of HR_max_.

### Intramuscular protein signaling

#### PKB/Akt

**Figure 2:**
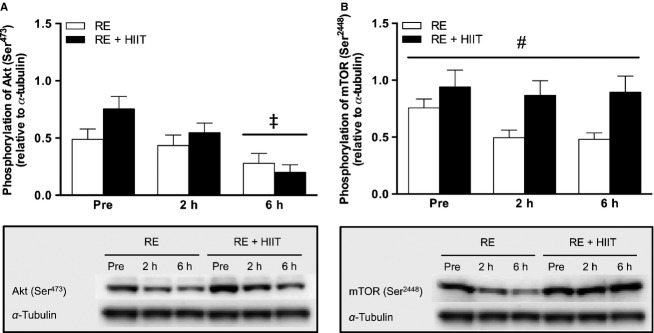
Phosphorylation of (A) Akt at Ser^473^, and (B) mTOR at Ser^2448^, before, 2 and 6 h after resistance exercise in both trials. RE, resistance exercise trial; RE + HIIT, resistance exercise and high-intensity interval training trial. Symbols denote differences revealed by a post hoc test when a main effect was observed. ^‡^*P *<* *0.05 versus Pre and 2 h; ^#^*P *<* *0.05 versus RE. Data presented as mean ± SE. *n *=* *10.

There was a main effect of time (*P *<* *0.05), but no main effect trial (*P *>* *0.05), or an interaction effect (*P *>* *0.05) for the phosphorylation state of Akt at Ser^473^. Post hoc analysis showed a decrease in Akt phosphorylation at 6 h after both exercise trials compared to baseline and 2 h (*P *<* *0.05, Fig.2A).

#### mTOR

There was a main effect of trial (*P *<* *0.05), but no effect of time (*P *>* *0.05), or an interaction effect (*P *>* *0.05) for the phosphorylation of mTOR at Ser^2448^. There was higher phosphorylation of mTOR at Ser^2448^ in RE + HIIT a compared to RE (*P *<* *0.05, Fig.2B).

#### eEF2, p70S6K, rpS6, 4E-BP1

There were no main effects of time (*P *>* *0.05), trial (*P *>* *0.05), or an interaction effect (*P *>* *0.05) for the phosphorylation state of eEF2 at Thr^56^, p70S6K at Thr^389^, rpS6 at Ser^235/236^, and 4E-BP1 at Thr^37/46^.

### Intramuscular mRNA expression

#### PGC-1*α*

**Figure 3:**
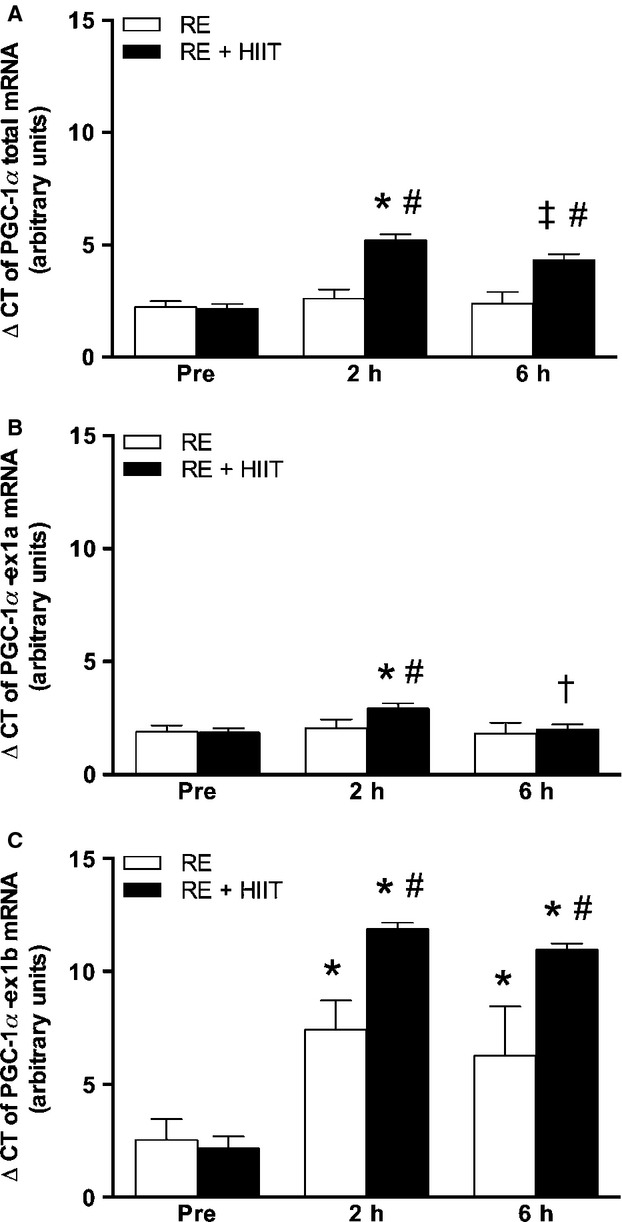
mRNA expression of (A) PGC-1*α* total, (B) PGC-1*α*-ex1a, and (C) PGC-1*α*-ex1b, before, 2 and 6 h after resistance exercise in both trials. RE, resistance exercise trial; RE + HIIT, resistance exercise and high-intensity interval training trial. Symbols denote differences revealed by a post hoc test when an interaction effect was observed. *P <* *0.05 versus Pre; ^†^*P *<* *0.05 versus 2 h; ^‡^*P *<* *0.05 versus Pre and 2 h; ^#^*P *<* *0.05 versus RE. Data presented as mean ± SE. *n *=* *10.

There were main effects of trial (*P *<* *0.05), and time (*P *<* *0.05), and an interaction effect (*P *<* *0.05) for the expression of total PGC-1*α*. Post hoc analysis revealed higher expression levels of total PGC-1*α* mRNA at 2 and 6 h in RE + HIIT compared to RE (*P *<* *0.05). In RE + HIIT, total PGC-1*α* mRNA was elevated, 8.2-fold, above baseline at 2 h (*P *<* *0.05) and remained 4.5-fold above baseline at 6 h (*P *<* *0.05, Fig.3A).

The expression of PGC-1*α*-ex1a mRNA showed a main effect of time (*P *<* *0.05), and an interaction effect (*P *<* *0.05), but no main effect of trial (*P *>* *0.05). Expression of PGC-1*α*-ex1a mRNA was 2.1-fold higher at 2 h compared to baseline in RE + HIIT (*P *<* *0.05) but had returned to baseline at 6 h (*P *>* *0.05). Conversely, the expression of PGC-1*α*-ex1a mRNA remained unchanged over time for RE (Fig.3B).

The expression of PGC-1*α*-ex1b mRNA showed main effects of time (*P *>* *0.05), and trial (*P *>* *0.05), and an interaction effect (*P *>* *0.05). The increase (*P *<* *0.05) at 2 and 6 h compared to baseline for both exercise trials was greater in RE + HIIT at 2 h (∼840-fold versus ∼25-fold in RE) and 6 h (∼450-fold vs. ∼15-fold in RE) (Fig.3C).

#### Myostatin

**Figure 4:**
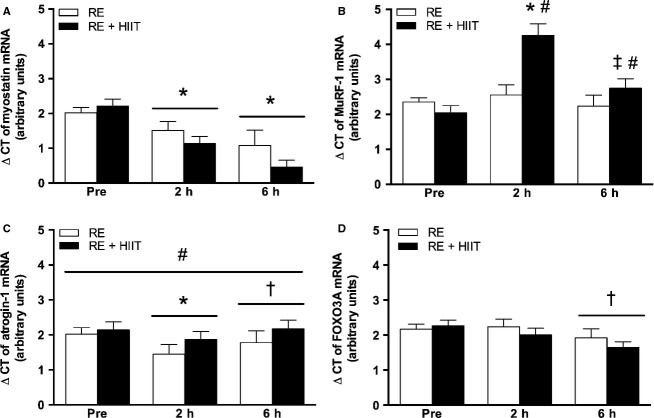
mRNA expression of (A) myostatin, (B) MuRF-1, (C) atrogin-1, and (D) FOXO3A, before, 2 and 6 h after resistance exercise in both trials. RE, resistance exercise trial; RE + HIIT, resistance exercise and high-intensity interval training trial. Symbols above lines denote differences revealed by a post hoc test when a main effect was observed. Symbols without lines denote differences revealed by a post hoc test when an interaction effect was observed. *P <* *0.05 versus Pre; ^†^*P *<* *0.05 versus 2 h; ^‡^*P *<* *0.05 versus Pre and 2 h; ^#^*P *<* *0.05 versus RE. Data presented as mean ± SE. *n *=* *10.

There was a main effect of time (*P *<* *0.05), but no effect of trial (*P *>* *0.05), or an interaction effect (*P *>* *0.05) for the expression of myostatin mRNA. Myostatin expression was downregulated compared to baseline at 2 h (∼1.7-fold, *P *<* *0.05) and 6 h (∼2.5-fold, *P *<* *0.05) following both exercise protocols (Fig.4A).

#### Proteolytic markers

There were main effects for time (*P *<* *0.05), and trial (*P *<* *0.05), and an interaction effect (*P *<* *0.05) for the expression of MuRF-1 mRNA. In RE + HIIT, MuRF-1 mRNA was elevated, 4.6-fold, above baseline at 2 h (*P *<* *0.05) and remained 1.6-fold above baseline at 6 h (*P *<* *0.05). Conversely, MuRF-1 was unaffected across time after RE (*P *>* *0.05, Fig.4B). There were main effects of time (*P *<* *0.05), and trial (*P *<* *0.05), but no interaction effect (*P *>* *0.05) for the expression of atrogin-1 mRNA. RE + HIIT showed a significant overall higher expression level of atrogin-1 mRNA compared to RE (*P *<* *0.05). Atrogin-1 mRNA expression showed a downregulation at 2 h compared to baseline and 6 h, irrespective of trial (*P *<* *0.05, Fig.4C).

There was a main effect of time (*P *<* *0.05), but no main effect of trial (*P *>* *0.05), or an interaction effect (*P *>* *0.05) for the expression of FOXO3A mRNA. Post hoc analysis showed that FOXO3A mRNA, regardless of exercise trial, was significantly lower at 6 h compared to 2 h (*P *<* *0.05) and a trend to be lower compared to baseline (*P *=* *0.053, Fig.4D).

#### IGF-1 and myogenic regulatory factors

**Figure 5:**
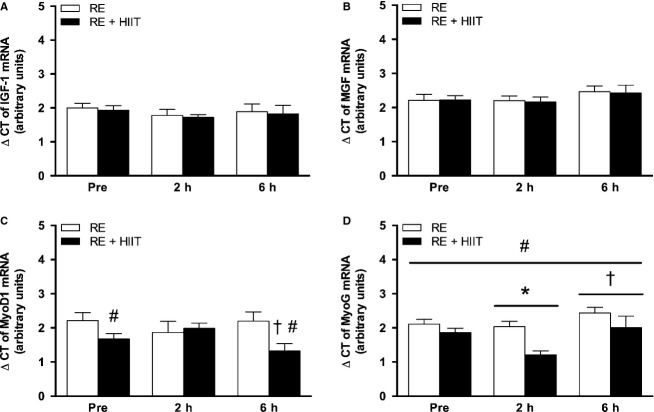
mRNA expression of (A) IGF-1, (B) MGF, (C) MyoD1, and (D) MyoG, before, 2 and 6 h after resistance exercise in both trials. RE, resistance exercise trial; RE + HIIT, resistance exercise and high-intensity interval training trial. Symbols above lines denote differences revealed by a post hoc test when a main effect was observed. Symbols without lines denote differences revealed by a post hoc test when an interaction effect was observed. *P <* *0.05 versus Pre; ^†^*P *<* *0.05 versus 2 h; ^#^*P *<* *0.05 versus RE. Data presented as mean ± SE. *n *=* *10.

There were no main effects of time (*P *>* *0.05), or trial (*P *>* *0.05), and no interaction effect (*P *>* *0.05) for the expression of IGF-1 (*P *>* *0.05; Fig.5A) and MGF mRNA (*P *>* *0.05; Fig.5B). There was an interaction effect (*P *<* *0.05), but no main effects of time (*P *>* *0.05), or trial (*P *>* *0.05) for the expression of MyoD1 mRNA. Further analysis of data revealed a minor but significant difference for MyoD1 mRNA expression at baseline (*P *<* *0.05) and 6 h (*P *<* *0.05) between exercise trials. At 6 h, MyoD1 mRNA expression showed a significant decrease compared to 2 h (*P *<* *0.05), but no significant differences compared to baseline in RE + HIIT (Fig.5C).

There were main effects of time (*P *<* *0.05), and trial (*P *<* *0.05), but no interaction effect (*P *>* *0.05) for the expression of MyoG mRNA. MyoG mRNA expression levels were lower in RE + HIIT compared to RE (*P *<* *0.05). Further analysis of the data showed a statistically significant decrease at 2 h (*P *<* *0.05) regardless of exercise trial, but by 6 h MyoG mRNA expression had returned to baseline (Fig.5D).

## Discussion

The development of a pragmatic exercise program, combining both resistance exercise and HIIT within a single session, does not compromise the RE-induced adaptations. This exercise protocol may help novice/untrained individuals meet the current physical activity guidelines by minimizing the number of exercise session per week. Moreover, RE + HIIT resulted in superior expression of total PGC-1*α*, PGC-1*α*-ex1a, and PGC-1*α*-ex1b over resistance exercise alone which may be indicative of enhanced mitochondrial biogenesis. Combining resistance training with endurance training has been shown to have greater improvements in both cardio-metabolic health and quality of life compared to single-mode exercise training in both healthy (Lundberg et al. 2013), and disease population groups (Zanuso et al. 2010). However, the use of traditional endurance exercise (Coffey et al. 2009b), or maximal sprints (Coffey et al. 2009a), following resistance exercise has been shown to negatively impact on the adaptation to the resistance exposure. The ASCM guidelines for physical activity recommends that adults should participant in 150 min of moderate physical activity per week, or 75 min of vigorous physical activity, plus at least two muscles strengthening sessions (∼30 min per session) per week (Garber et al. 2011). Here, our combination of RE + HIIT has the potential of combining both resistance and endurance training within a single exposure, whilst minimizing both the exercise time commitment (RE + HIIT vs. RE + moderate endurance exercise: 165 vs. 240 min per week), and the number of training sessions per week (concurrent RE + HIIT vs. individual exercise sessions: 3 vs. 7 sessions).

The experimental design compared the effects of concurrent RE + HIIT to a single session of RE on the basis that there is limited evidence (Leveritt et al. 1999; Wilson et al. 2012) to suggest that RE interferes with endurance adaptations and therefore a comparison to a single HIIT exposure was deemed redundant. In order to address the key question of this study “Are the benefits of the resistance exercise training lost if they are immediately followed by a session which stimulates the endurance adaptive pathway” the exercise trials were not evenly matched for workload, and the results could reflect the differences in work done between trials. An alternative experimental design whereby the concurrent exercise was compared to a prolonged resistance exercise session would not address the hypothesis.

In the current study, there were greater increases in PGC-1*α* expression following RE + HIIT compared to RE, but similar inhibition of proteolysis markers (FOXO3A and atrogin-1), suggesting a parallel boost in resistance- and endurance-type adaptation. The splice variant PGC-1*α*-ex1b, which has been shown to be associated with RE (Ruas et al. 2012, termed PGC-1*α*4) increased after RE in the current study but was further enhanced after RE + HIIT. HIIT protocols are associated with endurance adaptations and enhanced PGC-1*α* (Gibala et al. 2012), however, the evidence for an hypertrophic effect of HIIT is mixed with work showing both no change (Trapp et al. 2008; Nybo et al. 2010) and increases to lean mass (Heydari et al. 2012; Gillen et al. 2013). The respective increases in PGC-1*α*-ex1b expression at 2 and 6 h for RE and RE + HIIT, reported here suggest that the splice variants both respond in a similar manner, reflecting changes to total PGC-1*α*. It is worth noting that while the primers used in the current study were not able to distinguish between truncated and nontruncated forms, they did identify different splice variances in exon 1 (alternative and proximal promoters), as suggested by Ruas et al. (2012). However, whether PGC-1*α*-ex1b (truncated and/or nontruncated) plays a role in skeletal muscle hypertrophy cannot be confirmed.

Evidence drawn from animal studies suggests an exercise intensity-dependent increase in PGC-1*α* splice variants (Tadaishi et al. 2011), with PGC-1*α*-ex1a expression only increasing after high-intensity exercise. In the present study, only RE + HIIT resulted in a significant increase in PGC-1*α*-ex1a, which may have in part been due to the high-intensity *per se* of HIIT. Similarly, this may explain the exaggerated increase in PGC-1*α*-ex1b reported in RE + HIIT. However, further work is required to directly compare concurrent RE + HIIT to endurance only exercise at different intensities. It could be speculated from the work of Ruas et al. (2012) that the superior increase in PGC-1*α*-ex1b following RE + HIIT may result in greater skeletal muscle hypertrophy following training. However, Lundberg et al. (2014), recently disputed evidence for a truncated PGC-1*α* splice variant in the regulation of muscle hypertrophy. This group investigated 5 weeks of either resistance training or a combination of aerobic and resistance training (separated by 6 h); truncated PGC-1*α* increased regardless of exercise mode. Furthermore, there were no correlations found between PGC-1*α* splice variants and muscle size or strength (Lundberg et al. 2014). Concurrent training increased both alternative (exon 1b) and proximal (exon 1a) promoter transcripts, whereas resistance training only increased the alternative promoter (exon 1b) transcript (truncated and nontruncated forms) (Lundberg et al. 2014). Likewise, while the present study found only an increase in PGC-1*α*-ex1b in RE, the larger increase in RE + HIIT would suggest that HIIT has no negative impact. It remains unclear whether PGC-1*α*-ex1b (truncated and/or nontruncated) is preferentially expressed in response to resistance exercise; thus further research is required to determine if PGC-1*α*-ex1b has any relevance in the regulation of skeletal muscle hypertrophy.

A link between PGC-1*α* and the FOXO family has been implicated in the regulation of muscle protein degradation (Sandri et al. 2006). MuRF-1 and atrogin-1 are two muscle-specific E3 ubiquitin ligases that have been associated with atrophying muscles (Foletta et al. 2011), sharing the same transcription factor, FOXO3A (Sandri et al. 2004). In the current study, regardless of exercise there was a decrease in atrogin-1 expression at 2 h. Feeding prior to exercise may have resulted in the downregulation of atrogin-1 expression (Borgenvik et al. 2012), causing only minor changes in atrogin-1 expression levels and closely resembling that of FOXO3A (Louis et al. 2007). In contrast, MuRF-1 mRNA expression showed a significant increase at 2 and 6 h in RE + HIIT compared to RE. The increase in MuRF-1 with RE + HIIT could be reflective of contractile protein repair, with similar responses evident following resistance exercise (Louis et al. 2007; Borgenvik et al. 2012), endurance exercise (Louis et al. 2007; Harber et al. 2010), and when performed together (Lundberg et al. 2012; Camera et al. 2015). Collectively, the present data suggest that concurrent exercise also shows little or no evidence of interference in protein breakdown via the ubiquitin-proteasome pathway above that of the required contractile protein repair.

In an attempt to optimize the anabolic response and mimic real-life training, we examined participants in a fed state rather than the fasted state commonly used in assessing the effects of concurrent exercise (Baar and Esser 1999; Coffey et al. 2009a,b). It could be speculated that prior feeding may have masked an anabolic stimulus following the exercise (Deldicque et al. 2009). Nevertheless, it can be interpreted that concurrent RE + HIIT did not cause any anabolic interference via mTOR signaling pathways, therefore supporting previous work with concurrent resistance exercise and traditional endurance exercise protocols (Donges et al. 2012; Lundberg et al. 2012). Furthermore, Gillen et al. (2013) have shown equal improvements in body composition, 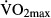, and muscle oxidative capacity when HIIT was conducted in a fed or fasted state. This highlights that regardless of when food is ingested there is no decrease in the sensitivity to detect changes in these adaptations to HIIT. The purpose of the study was to examine the “real-life” situations where most individuals will eat prior to training, and although this design may have led to elevated levels of phosphorylation and gene expression in the baseline biopsy the study highlights that concurrent RE + HIIT following feeding does not result in an acute molecular interference to resistance exercise (Donges et al. 2012; Lundberg et al. 2012).

Results of IGF-1 and MGF mRNA showed no changes in expression following either exercise trial. RE + HIIT appears to have no negative effect on IGF-1 and/or MGF mRNA expression compared to RE alone. Although the exact importance of IGF-1 and/or MGF remains unclear, previous studies of resistance exercise have shown mixed results of IGF-1 mRNA with increases (Bamman et al. 2001; Petrella et al. 2006), decreases (Psilander et al. 2003; Bickel and Slade 2005), and no change (Bickel et al. 2003; Hameed et al. 2003) being reported. In addition, two key regulators involved in the determination and differentiation of muscle cells, are MyoD and MyoG (Perry and Rudnick 2000). Both endurance exercise and resistance exercise have been shown to increase the expression of MyoD1 (Yang et al. 2005), with a similar increase in expression following concurrent exercise (Coffey et al. 2009a,b). In the current study, there was little or no change in MyoD1 expression regardless of exercise, which could have been the result of feeding prior to exercise (Deldicque et al. 2009). Myostatin is a negative regulator of muscle growth (McPherron et al. 1997), predominantly through the suppression of muscle protein synthesis (Welle et al. 2009), and has been shown to decrease in response to both resistance and endurance exercise completed alone or in combination (Louis et al. 2007; Lundberg et al. 2012). Here, mRNA expression of myostatin was reduced to similar extents in both RE and RE + HIIT, which suggests that the exercise-induced downregulation of myostatin and increase in MyoD1 is not driven by exercise-mode specificity but rather the contractile activity. Thus, these data further demonstrate no acute interference in molecular events leading to muscle growth adaptations with concurrent exercise.

In mimicking real-life training the postprandial state of the participants may explain why there was no change in the molecular event of MyoG expression following resistance exercise. However, it was shown that regardless of time MyoG expression in RE + HIIT was lower than RE. In addition, overall MyoG expression was lower at 2 h, regardless of the exercise trial, which appears to be driven mainly by the suppression in RE + HIIT. Although the mechanism for this finding is unclear, it could be speculated that RE + HIIT may have resulted in further suppression of MyoG over the feeding response in RE, which could lead to reduced satellite cell activation, proliferation, and differentiation, and therefore ultimately reduced hypertrophy. It has been reported that satellite cell activity is suppressed 96 h after both endurance exercise (−7%) and concurrent exercise (−8%; RE followed by endurance exercise), compared to an increase shown following resistance exercise alone (46%) (Babcock et al. 2012). This suggests that concurrent exercise may attenuate the satellite cell response to resistance exercise, which is essential for maximizing muscle growth, and may therefore hinder maximal hypertrophy. Clearly, before any conclusions can be drawn further research is required to determine the effect of concurrent exercise on the late-phase molecular responses following resistance exercise.

In summary, the current study, adopting real-life practices, demonstrates that concurrent resistance and high-intensity interval training performed in a fed state does not dampen the signaling arising from a single bout of resistance exercise.
